# Triangulation-Based Spatial Clustering for Adjacent Data With Heterogeneous Density

**DOI:** 10.1002/sam.70017

**Published:** 2025-04-18

**Authors:** Sihan Zhou, Daniel Vasiliu, Shi Qi, Guannan Wang

**Affiliations:** 1 Sloan School of Management, Massachusetts Institute of Technology, Cambridge, Massachusetts, USA; 2 Data Science Program, William & Mary, Williamsburg, Virginia, USA; 3 Department of Economics, William & Mary, Williamsburg, Virginia, USA; 4 Department of Mathematics, William & Mary, Williamsburg, Virginia, USA

**Keywords:** adjacent boundary, complex domain, DBSCAN, delaunay triangulation, density estimation

## Abstract

In diverse fields such as geography, meteorology, and economics, data often exhibit complex, nonlinear relationships, irregular structures, and are frequently collected over intricate domains. While effectively identifying regularly shaped clusters (e.g., ellipsoidal or spherical) within regular domains, traditional clustering algorithms often struggle with irregular cluster shapes, heterogeneous densities, noisy inter-cluster boundaries, and datasets spread across complex spatial domains. To address these challenges, we introduce a novel Density and Triangulation-based Clustering (DTC) framework, designed to excel in these complex scenarios through three key innovations: (1) density-based separation using an advanced density estimation method tailored for complex domains, (2) Delaunay triangulation-based spatial clustering, effectively managing nonlinear geometries and resolving adjacency issues, and (3) noise mitigation through proximity analysis leveraging nearest neighbors. The DTC framework uniquely integrates graph-based methods with robust density estimation methods, enabling it to handle cases where traditional algorithms fail. Extensive experiments on both synthetic and real-world datasets demonstrate its superior capability to identify nested and contiguous clusters with heterogeneous densities, even in the presence of noise and over complex domains. These findings underscore the practical applicability and versatility of DTC in extracting meaningful insights from challenging datasets.

## 1 | Introduction

Cluster analysis [1] is a key unsupervised learning technique that groups data points based on inherent patterns without the need for labeled training data. It plays an essential role in various disciplines, including geography [2], meteorology, and economics, where revealing hidden structures in data is critical. However, clustering is challenging when group information is not explicitly available. Spatial clustering, a specialized branch of cluster analysis, focuses on geospatial, remote sensing, and image data. The complexity of spatial data—marked by irregular shapes, varying densities, and distribution across intricate domains — often limits the performance of traditional clustering methods. Algorithms like K-means [3], for example, excel at detecting spherical clusters, but falter when dealing with non-spherical shapes or irregular data.

In response, Density-Based Clustering Algorithms (DBCLAs) have gained popularity due to their ability to identify clusters of arbitrary shapes and densities. DBSCAN [4] exemplifies this class of algorithms by defining clusters based on data point density within a specified radius. Subsequent advancements, such as Hierarchical DBSCAN (HDBSCAN) [5] and the Ordering Points to Identify the Clustering Structure (OPTICS) algorithm [6], incorporate hierarchical structures and reachability metrics to handle varying densities and reduce sensitivity to parameters. Other probabilistic approaches, including the Statistical Information Grid in Data Mining (STING) [7] and the Interactive Projected Clustering Algorithm (IPCLUS) [8], employ probabilistic models and kernel density estimation to manage irregular densities better.

Despite these advances, a key limitation of many existing DBCLAs is their reliance on density estimation methods that primarily use Euclidean distance. While Euclidean metrics can approximate density in simple, uniform domains, they often fail in more irregular spaces. Incorporating geodesic distances can improve accuracy, but is computationally expensive for large datasets and complex domains. This challenge motivates the development of new approaches that can efficiently and accurately handle density estimation in irregular settings, enabling more effective clustering in demanding scenarios.

**FIGURE 1 |:**
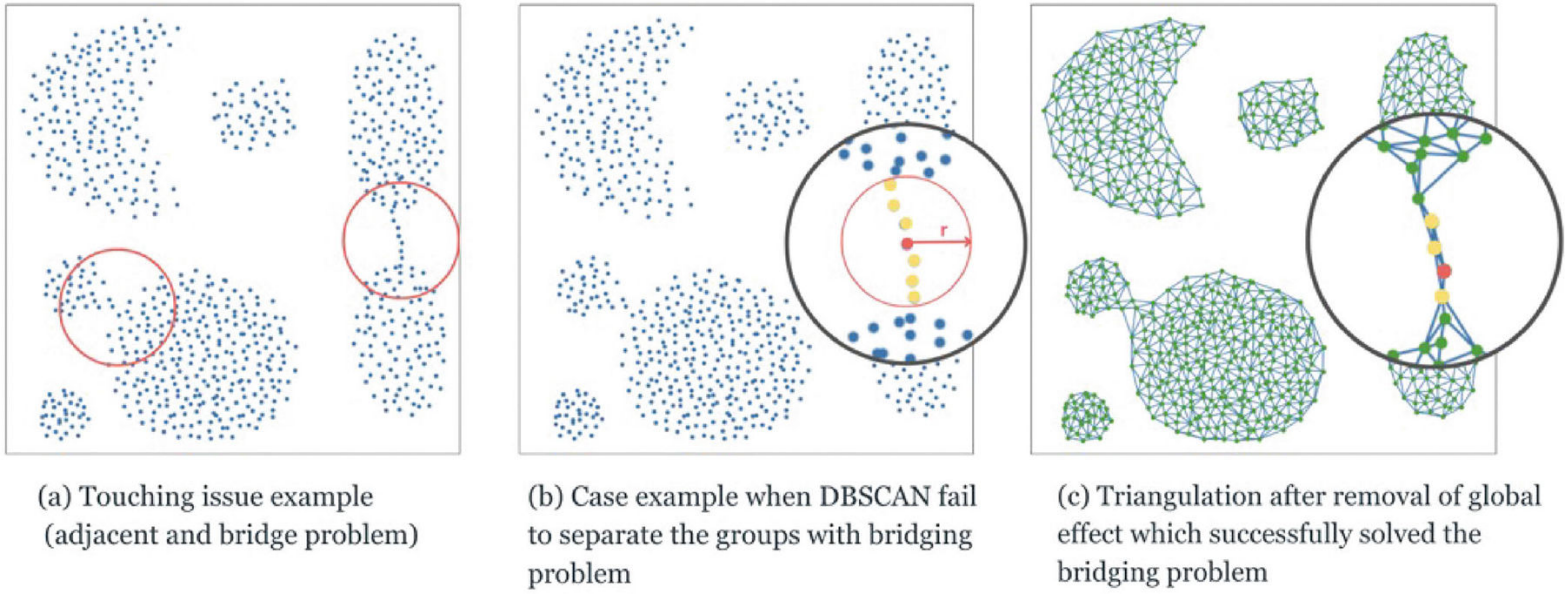
Illustration of the touching issue and the effectiveness of triangulation in solving this issue.

Another common shortcoming of current DBCLAs involves the “touching issue,” where closely spaced or adjacent clusters are difficult to separate (Figure 1a). Recent studies [9, 10] have shown that incorporating Delaunay triangulation can address this problem. By leveraging the triangulation’s max-min property [11], one can define more meaningful proximity relationships and effectively separate clusters connected by narrow links. Additionally, the “removal of global effect” concept [9] aids in discarding triangles that distort the intended shape, as illustrated in Figure 1b,c.

Still, these triangulation-based methods often struggle with highly irregular densities or nested clusters. To overcome such limitations and generalize clustering algorithms to accommodate arbitrary shapes, complex density relationships, and irregular domains, we introduce the Density and Triangulation-based Clustering (DTC) algorithm. DTC integrates Delaunay triangulation with an advanced density estimation strategy and the robust clustering framework of DBSCAN. This combination strikes a balance among accuracy, scalability, and adaptability. By using Delaunay triangulation, DTC captures intricate spatial relationships and adapts to irregular domains more effectively than Euclidean-based methods. Moreover, its innovative density estimation approach improves the precision of cluster detection across a wide range of densities and shapes. In doing so, DTC not only addresses the current limitations of clustering algorithms but also establishes itself as a powerful, generalizable tool for analyzing complex data.

This paper is organized as follows. Section 2 presents the methodology and toolkits employed in the DTC framework, including Delaunay triangulation, a novel density estimation method based on triangulation, a variant version of DBSCAN, and KNN for noise handling. Section 3 demonstrates the performance of the proposed DTC framework through simulations and real-world applications, including an analysis of PM_2.5_ pollution and clustering of FDIC’s bank branch data. The concluding section reviews DTC’s distinctive features and outlines potential future enhancements.

## 2 | Methodology and Implementation Details

**FIGURE 2 |:**
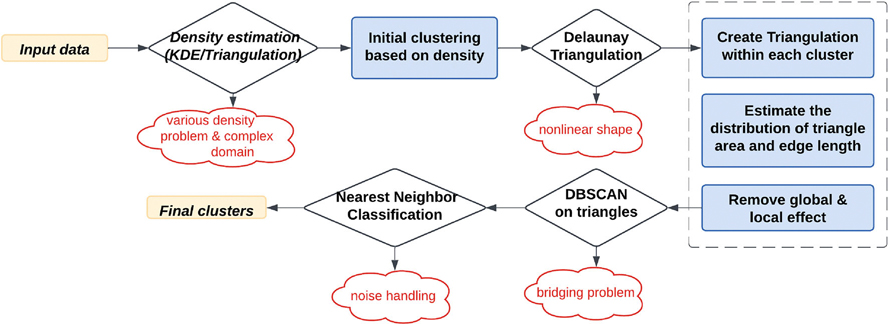
Flowchart of the density and triangulation-based clustering algorithm.

This section describes the methodology underlying the DTC algorithm, a novel framework designed to address challenges in clustering data with irregular shapes, heterogeneous densities, and complex domains. The DTC framework integrates advanced density estimation, triangulation-based clustering, and optimized data processing techniques, as illustrated in Figure 2. The following subsections provide an overview of the algorithm and detailed explanations of its key components.

### 2.1 | Overview of the Proposed Method

The new DTC algorithm introduces a series of innovations to overcome the limitations of traditional clustering methods. These

**ALGORITHM 1 |:** Density-based separation.

1:	**Input:** data points P with their corresponding kernel density D
2:	**Initialization:** ${𝓒}_{i}=\emptyset$, i=0, 𝓥=P, 𝓛=∅
3:	**while** 𝓥≠∅ **do**
4:	Find the point p∈𝓥 with the maximum kernel density in D and the closest point $p^{\prime }\in 𝓥$.
5:	Move p, $p^{\prime }$ from 𝓥 to 𝓒. Record the step size between p and $p^{\prime }$ as l in 𝓛.
6:	Find the next closest point $p^{\prime }\in 𝓥$ to the set 𝓒 and calculate the step size l
7:	**if** l>(μ(𝓛)+cσ(𝓛)) or $l>Q_{3}(𝓛)+c^{\prime }IQR(𝓛)$ **then**
8:	i+=1
9:	**else**
10:	Move $p^{\prime }$ from 𝓥 to 𝓒. Record the corresponding l into 𝓛.
11:	**end if**
12:	**end while**

enhancements are specifically tailored to address challenges such as irregular cluster shapes, heterogeneous density distributions, and adjacency issues. The key components of the proposed method are outlined as follows.

#### Advanced Density Estimation.

Unlike earlier triangulation-based approaches, which primarily rely on triangle features to separate clusters, the DTC algorithm incorporates a novel triangulation-based density estimation technique. This method accurately captures the underlying spatial distribution of data over any domain, including those with complex or irregular boundaries. By leveraging this innovative density estimation framework, the algorithm improves precision in identifying clusters with heterogeneous densities, significantly enhancing its applicability to challenging datasets.

#### Density-Based Partitioning.

The density estimation framework is followed by a density-based partitioning mechanism (illustrated in Algorithm 1). This approach divides the dataset into distinct regions based on calculated density levels, ensuring that areas with varying densities are effectively separated before clustering. This partitioning process provides a robust foundation for subsequent steps and effectively resolves issues such as closely spaced clusters.

#### Delaunay Triangulations and Refinement.

Delaunay triangulations are employed within each partitioned region to refine cluster boundaries. The algorithm addresses the “global effect” by systematically removing triangles with disproportionately large edge lengths or areas based on statistical distributions. It also mitigates the “local effect” by excluding outlying edges connected to individual vertices. These refinements enhance the delineation of clusters with non-linear geometries, ensuring an accurate representation of the underlying data structure.

#### Modified DBSCAN and Noise Reassignment.

The DTC algorithm integrates a modified version of DBSCAN, which replaces the conventional radius-based clustering approach with a vertex-neighbor count derived from triangulation. This adaptation resolves adjacency issues, including bridging phenomena, and improves the algorithm’s ability to navigate complex spatial relationships.

To further enhance robustness, noise points identified in the DBSCAN phase are reassigned using K-Nearest Neighbors (KNN). This step ensures accurate classification of boundary points, thereby refining the final clustering results.

By combining advanced density estimation, innovative partitioning, efficient triangulation-based clustering, and robust noise handling, the DTC algorithm achieves high accuracy, scalability, and adaptability. This cohesive framework addresses the limitations of existing clustering methods and delivers superior performance across diverse applications.

### 2.2 | Delaunay Triangulations

Delaunay triangulation [12] is a widely used geometric technique for approximating spatial domains. It constructs triangles such that no data point lies inside the circumcircle of any triangle. This property ensures the maximization of the minimum angles within the triangles, effectively minimizing the occurrence of excessively narrow shapes. As a result, Delaunay triangulation provides a well-balanced and uniform representation of spatial relationships, making it particularly suited for complex and irregular domains.

Delaunay triangulations can be constructed using various software tools, including the Triangulation [13] package in R, the Distmesh program [14] for Matlab, and the scipy.spatial.Delaunay [15] package in Python. These implementations enable efficient triangulation of large datasets, making them suitable for a wide range of research applications.

In the DTC framework, Delaunay triangulations serve as the basis for density estimation and cluster refinement. Their flexibility allows the algorithm to handle complex spatial domains, such as those with irregular geometries or nested clusters.

### 2.3 | Triangulation Based Density Estimation

Density estimation plays a crucial role in determining the probability distribution of a random variable from a set of data points. It is especially important for resolving the “cluster touching” problem, where clusters of varying densities might be incorrectly identified as a single cluster without proper separation. This issue is particularly relevant in complex datasets with irregular or nested structures. While triangulation-based methods offer some separation, density estimation provides a more nuanced approach, effectively distinguishing adjacent clusters. Incorporating density estimation enhances the generalizability of the DTC algorithm, enabling it to capture relationships between data points regardless of variations in cluster shape or density. However, traditional density estimation methods, such as Kernel Density Estimation (KDE) [16], which rely on Euclidean distances, often struggle with edge effects near domain boundaries. This leads to biased estimates and reduced accuracy, particularly when dealing with complex spatial domains featuring irregular shapes, interior holes, or sharp boundaries. To address these limitations, this article proposes a novel triangulation-based density estimation method designed for greater flexibility and adaptability to such complex geometries.

**FIGURE 3 |:**
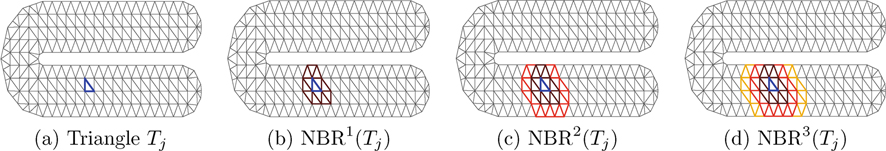
An illustration of a triangle and its neighbors.

To approximate a complex domain Ω, we employ triangulation, denoted as $\text{Δ}=\cup_{j=1}^{N}T_{j}$, where $T_{j}$ represents a collection of triangles such that each triangle shares edges or vertices with its neighbors. This approach provides a flexible representation of the domain’s geometry. An example of such triangulation of a horseshoe domain is illustrated in Figure 3. At any point $u\in T_{j}$, the data density can be estimated using:

$$\hat{f}(u)=\sum_{j=1}^{N}I\left(u\in T_{j}\right)\frac{n_{j}a_{j}}{nA}$$

where, $n_{j}$ is the number of data points within triangle $T_{j}$, n is the total number of data points, A is the overall area of the domain, and $a_{j}$ is the area of $T_{j}$. This estimator offers a basic approximation of the data distribution over the domain.

However, for datasets with low sample sizes, this approach can yield inaccurate or near-zero density estimates in triangles containing few or no data points. To overcome this limitation, we enhance the density estimator by incorporating information from neighboring triangles. Specifically, for a given triangulation Δ, we define the lth layer of neighboring triangles (NBR) for a triangle $T_{j}$ recursively as $\omega_{j}^{𝜄}=\text{NB}{\text{R}}^{𝜄}\left(T_{j}\right)\equiv \cup \left\{T\in \text{Δ}:T\;\cap \text{NB}{\text{R}}^{𝜄-1}\left(\omega_{j}\right)\neq \emptyset \right\}$, with the base case $\omega_{j}^{0}=T_{j}$. Figure 3 provides an example of a sub-region within a horseshoe domain, illustrating the different layers of neighbors.

Using this framework, we define the refined density estimator as:

$$\hat{f}(u)=\sum_{j=1}^{N}I\left(u\in T_{j}\right)\frac{N_{j}A_{j}}{nA}$$

where, $N_{j}$ is the total number of data points within the neighborhood of $T_{j}$, and $A_{j}$ represents the total area of the neighborhood. This refined estimator significantly improves accuracy, particularly for sparse datasets, by leveraging spatially proximate data to mitigate sparsity-induced inaccuracies. The incorporation of this triangulation-based density estimation method into our DTC algorithm has proven instrumental in enhancing the robustness of our clustering approach, especially for data distributed over complex domains.

### 2.4 | Density-Based Separation

Density estimation is also particularly pronounced in resolving the issue of “cluster touching” in datasets with varying densities. In such scenarios, clusters with different densities may be erroneously perceived as singular entities without the intricate separation afforded by density estimation. This technique becomes increasingly vital as datasets evolve in complexity, exhibiting irregular and nested structures. Density estimation facilitates the nuanced separation of adjacent clusters, a task that may not be fully achievable through triangulation-based methods alone. By incorporating density estimation, the DTC algorithm attains a level of generalizability, effectively capturing relationships between data points irrespective of the variations in shape or density present within the dataset.

Let 𝓟 be all the data points, ${𝓒}_{i}$ denotes the set of points that are put into the same cluster i, and 𝓥 denotes the remaining points. Initially, we set 𝓒=∅, 𝓥=𝓟. We first estimate the density for each data point. Among all the data points, we find point p∈𝓥 with the highest density and put it in 𝓒. Then we find another point $p^{\prime }\in 𝓥$ that is closest to p, and move $p^{\prime }$ into the set 𝓒, record the corresponding step size s. Then, within 𝓥, we look for the next point closest to any point in S but only move it into the cluster if the new step size is not significantly different compared to the distribution of all the past step sizes. We repeat the process until v=∅.

**FIGURE 4 |:**
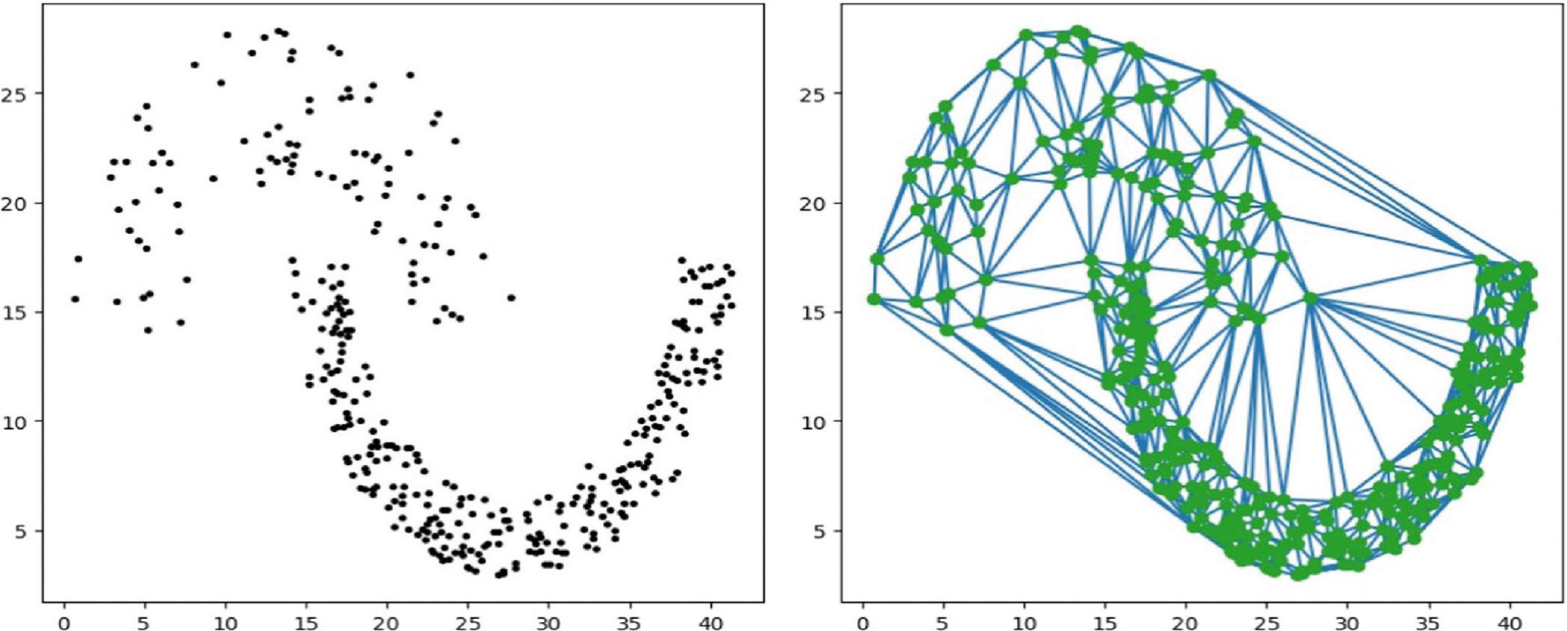
An example of data set with various density.

**FIGURE 5 |:**
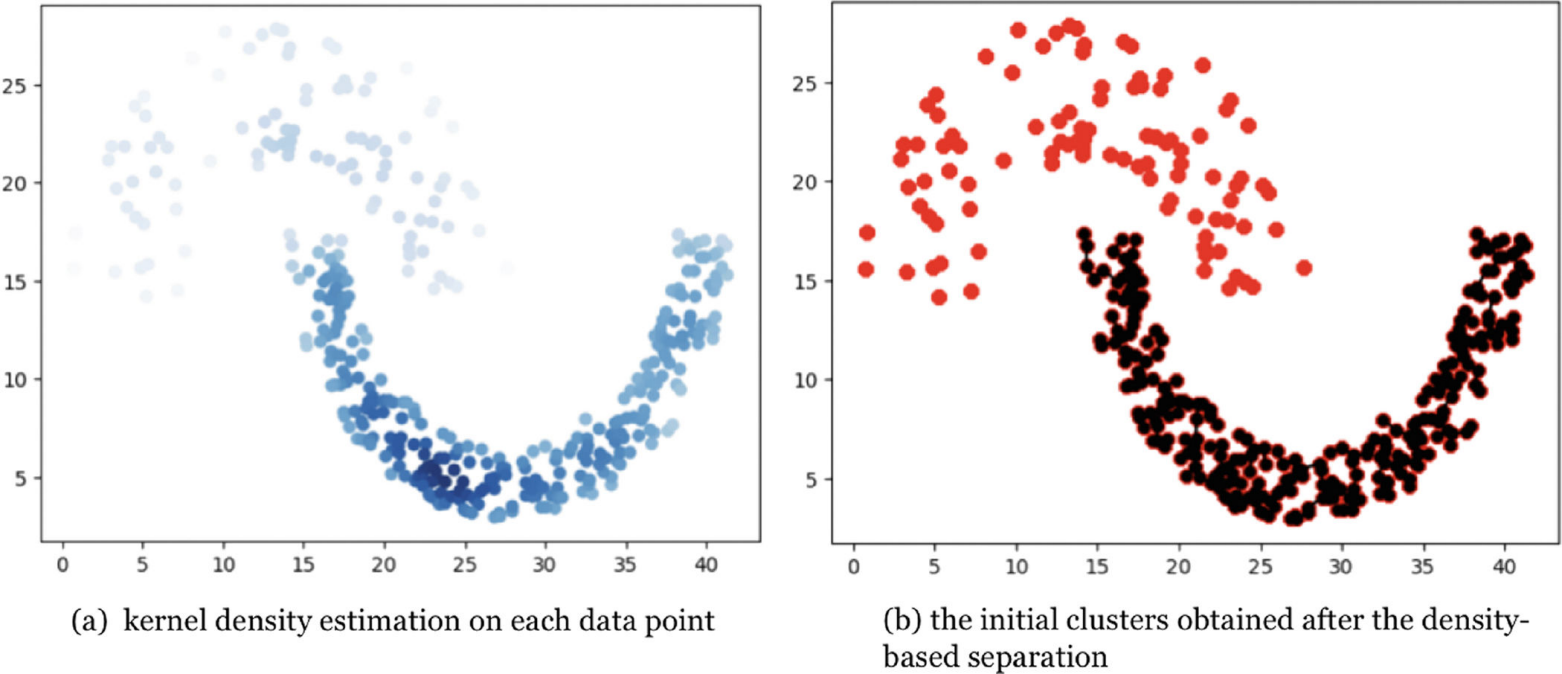
Perform density-based partitioning on the same data set.

Figure 4 illustrates a typical example of two closely positioned clusters with varying densities, posing challenges for traditional methods to distinguish them. Considering Figure 5a, it becomes evident that the two moon-shaped data groups warrant classification into distinct clusters, primarily owing to the discernible differences in their density profiles. The implementation of the aforementioned algorithm facilitates this distinction, enabling the identification of clusters that exhibit varying density characteristics. This capability surpasses what could be achieved through methods that exclusively depend on local proximity relationships, highlighting the efficacy of the algorithm in distinguishing between clusters with heterogeneous density distributions.

**ALGORITHM 2 |:** Triangulation-based DBSCAN.

1:	**Input:** data points X, a set of triangles T, and the hyperparamter *minPts*.
2:	**Initialization:** Create a stack 𝓒=∅, a list of unvisited points 𝓥=X. Set cluster index i=0.
3:	**while** 𝓥=∅ **do**
4:	Randomly choose a point from 𝓥 and add it into 𝓒.
5:	**while** 𝓒≠∅ **do**
6:	Pop a point p from 𝓒, add all the vertices connected to p into 𝓒.
7:	Count the number vertices connected to p, compare it with *minPts*.
8:	**if** number of vertices > *minPts* **then**
9:	mark p as a member of cluster i
10:	**else**
11:	mark p as a noise
12:	**end if**
13:	**end while**
14:	i+=1
15:	**end while**

### 2.5 | Removal of Global and Local Effect

Our methodology commences with a global estimation of the distribution of both the area and edge length across all triangulated structures. Leveraging the inherent attributes of triangulation, it becomes feasible to exclude triangles that exhibit excessively long edges or disproportionately large areas. This exclusion is crucial for unveiling potential clusters within the data. In scenarios where data points within a cluster are relatively uniformly distributed, the resultant triangulation predominantly consists of triangles that closely approximate equilateral forms. Consequently, it becomes pertinent to eliminate edges that are outliers in the context of their connection to a single vertex. This process constitutes a comprehensive pre-processing step in our clustering algorithm, addressing both global and local anomalies.

**FIGURE 6 |:**
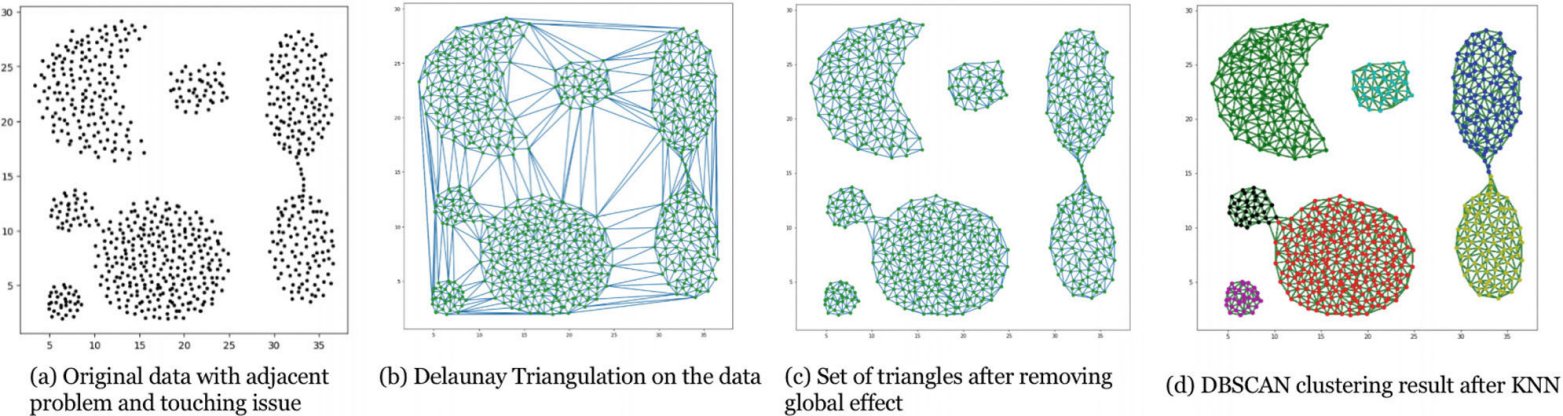
Example of using triangulation-based DBSCAN to solve adjacent problems.

Figure 6c shows the resulting set of triangles after we remove the global and local effects, followed by the distribution of areas and edge length of triangles in (b). Now, we could perform DBSCAN by choosing the minimum points, a value of points required to form a cluster, as the hyperparameter. See Algorithm 2 for a detailed procedure description.

### 2.6 | Relabeling of Noise

Up to this juncture in our analysis, we have achieved an approximately precise clustering outcome by integrating the density estimation method, Delaunay triangulation, and DBSCAN. However, a notable limitation arises from DBSCAN’s hyperparameter, specifically “minPts” (minimum points), which tends to result in the erroneous classification of data points near cluster boundaries as noise. To address this issue, we subsequently apply a K-Nearest Neighbors (KNN) classification algorithm. This additional step is instrumental in reassessing the data points previously designated as noise within the clustering framework, thereby refining the overall accuracy of the clustering results.

## 3 | Numerical Experiments

To evaluate the performance of the proposed DTC algorithm, we conducted extensive numerical experiments using a range of benchmark synthetic datasets. These experiments assess the DTC algorithm’s ability to handle irregular cluster shapes, heterogeneous densities, and adjacency issues. The results are compared with several established clustering methods, including K-Means, DBSCAN, HDBSCAN, and OPTICS.

The proposed algorithm was applied to widely used synthetic datasets, including the “Aggregation” dataset, “A.K. Jain’s Toy Problem” dataset, and “Zahn’s Compound” dataset in [17], as well as classical benchmarks from the scikit-learn library (e.g., Aniso, Varied, and Noisy Circles datasets) and a complex horseshoe-shaped domain dataset [18]. These datasets pose diverse challenges, such as irregular cluster shapes, adjacency, and nested clusters with varying densities.

### Aggregation Dataset.

**FIGURE 7 |:**
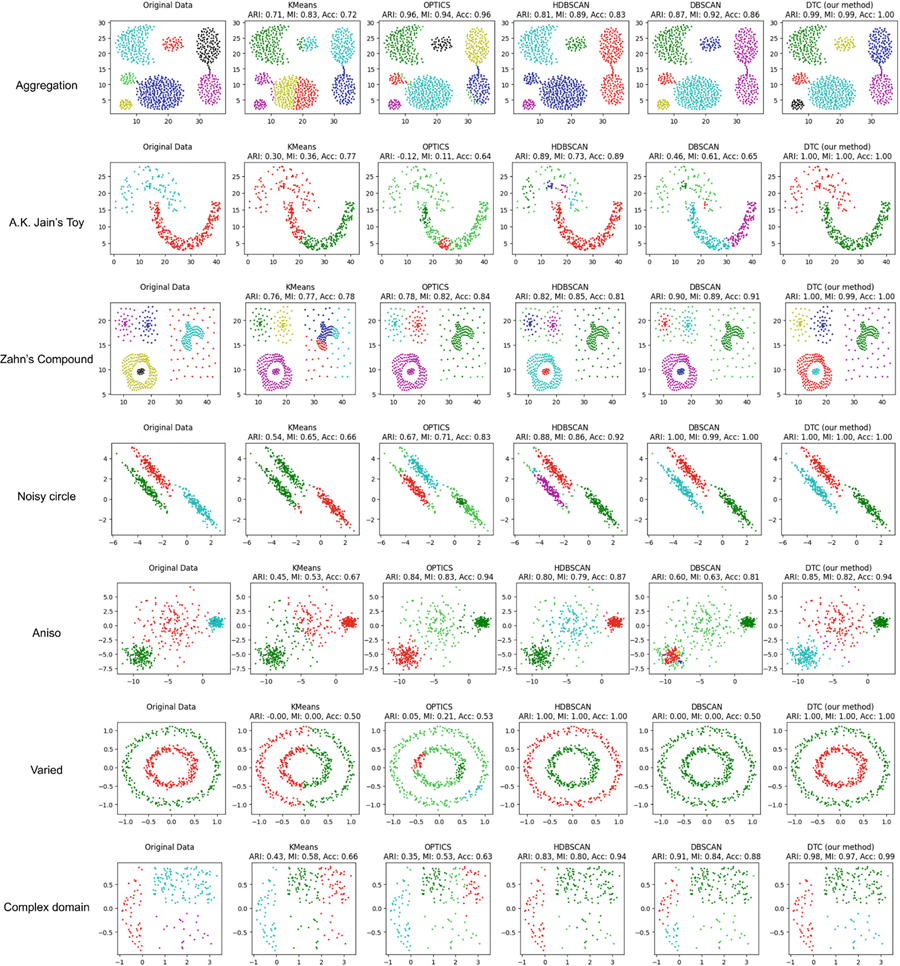
Comparison of the performance on the synthetic data set.

The Aggregation dataset, shown in Figure 7, is a classical example featuring adjacent and bridging clusters with nonlinear structures. Traditional algorithms, such as K-Means, struggle with the nonlinearity of the cluster shapes, and density-based methods often fail to distinguish adjacent clusters. In contrast, our DTC methodology initially eliminates relatively long edges, which enables a more precise separation of touching clusters and bridges, especially after the global and local removal of extraneous triangles in preparation for DBSCAN, as shown in Figure 6. The resulting clusters demonstrate DTC’s superior capability in preserving cluster integrity while addressing adjacency issues.

### A.K. Jain’s Toy Problem.

This dataset consists of two moon-shaped clusters with different densities in close proximity. Traditional methods, including DBSCAN and HDBSCAN, either fail to preserve the shape of the clusters or over-segment the data. The DTC algorithm, however, resolves these challenges by integrating density estimation and triangulation-based clustering, achieving nearly perfect accuracy, as demonstrated in Table 1 and Figure 6.

### Zahn’s Compound Dataset.

Zahn’s Compound dataset presents nested and adjacent clusters with varied densities. The DTC algorithm excels by leveraging precise density estimation and effective separation by triangulation-based DBSCAN, outperforming traditional methods that struggle with the complexity of the dataset. The DTC algorithm adeptly separates closely nested groups, even in the presence of noise.

### Performance Across Scikit-learn Datasets.

For the scikit-learn benchmarks, DTC consistently outperformed traditional methods on various datasets.

- *Aniso dataset*: DTC and DBSCAN both identify elongated clusters accurately, showcasing their ability to adapt to non-spherical shapes.
- *Varied dataset*: Shifting to a more complicated patterned dataset like Varied, DTC effectively handles clusters with differing densities, unlike DBSCAN, which relies on fixed density thresholds.
- *Noisy Circles dataset*: DTC captures circular structures, a task where K-Means fails and DBSCAN underperforms due to uniform density assumptions.

These results highlight DTC’s ability to model complex, non-linear, and non-uniform cluster structures, making it significantly more versatile than traditional methods.

### Data over Horseshoe Domain.

The horseshoe domain dataset is derived from a classic example introduced in [18], designed to evaluate the performance of clustering algorithms on data distributed over complex domains. This dataset features a spatially intricate horseshoe-shaped domain characterized by sharp concavities and a disjointed configuration that mimics real-world phenomena, such as geographical regions. Figure 7 illustrates this domain and the underlying data distribution. Traditional methods, such as K-Means and DBSCAN, face significant challenges in delineating boundaries in such irregular domains. Our DTC method overcomes these limitations by employing triangulation-based density estimation and global and local effect removal, achieving superior accuracy and interpretability.

## 4 | Real Life Applications

### 4.1 | U.S. PM_2.5_ Analysis

To further examine the applicability of the proposed DTC algorithm to real data, we applied the proposed algorithm to meteorological data. We used the daily mean surface concentrations of total PM_2.5_ for the year 2011 obtained from the United States Environmental Protection Agency. Among all the observed sites, we selected the locations where the corresponding PM_2.5_ value is greater than 12 *μ*g/m^3^, the threshold value given by the U.S. Environmental Protection Agency (EPA) to differentiate good and potentially harmful air quality.

**FIGURE 8 |:**
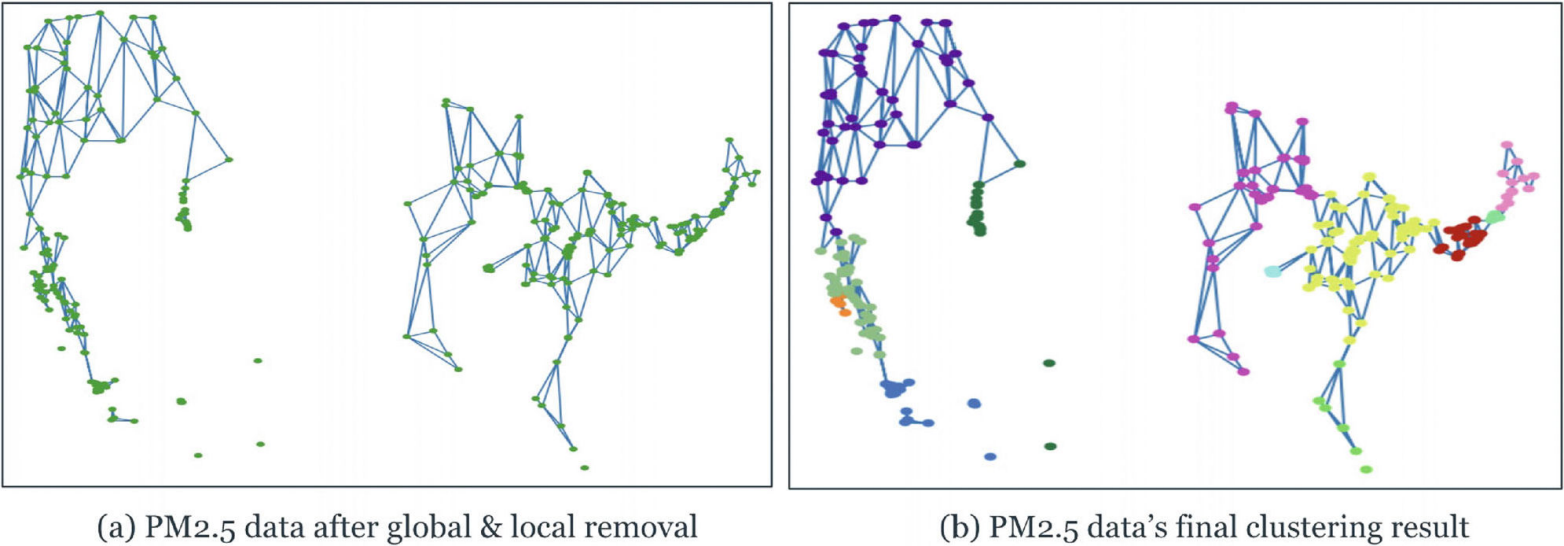
Apply the triangulation algorithm to PM_2.5_ data.

**FIGURE 9 |:**
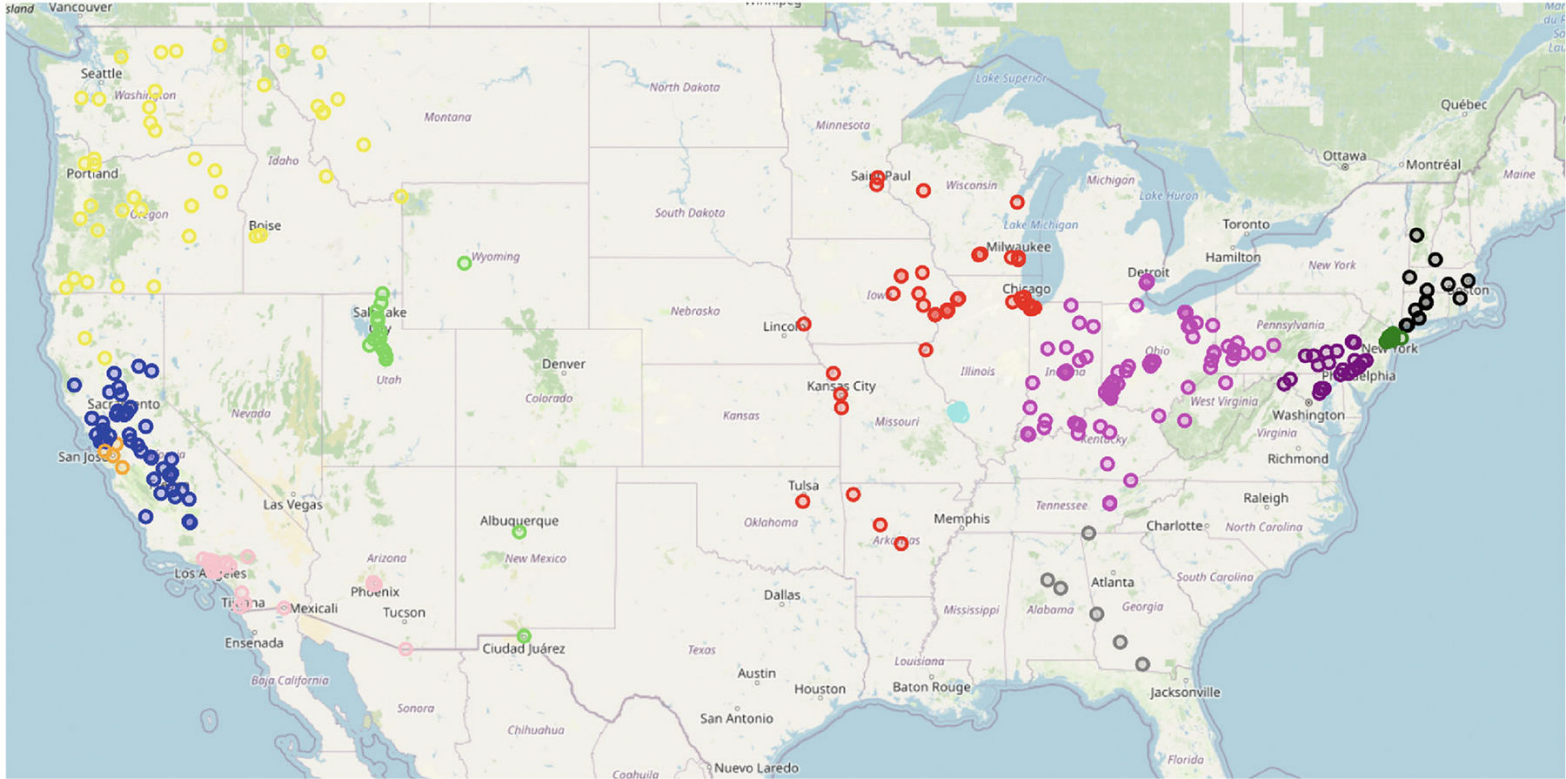
Clustering result on the 2011’s PM_2.5_ data in the United States.

Upon implementing our algorithm as depicted in Figure 8, the clustering results from Figure 9 demonstrate that the nonlinear-shaped PM_2.5_ spatial data points are separated into 12 clusters, providing insightful information on the air quality across various regions in the U.S. The dark green, purple, cyan, and orange clusters specifically pinpoint major urban centers, including New York, Philadelphia, San Jose, and St Louis. Despite these cities being represented by closely situated data points that could be prone to misclassification, our algorithm effectively differentiates them.

**FIGURE 10 |:**
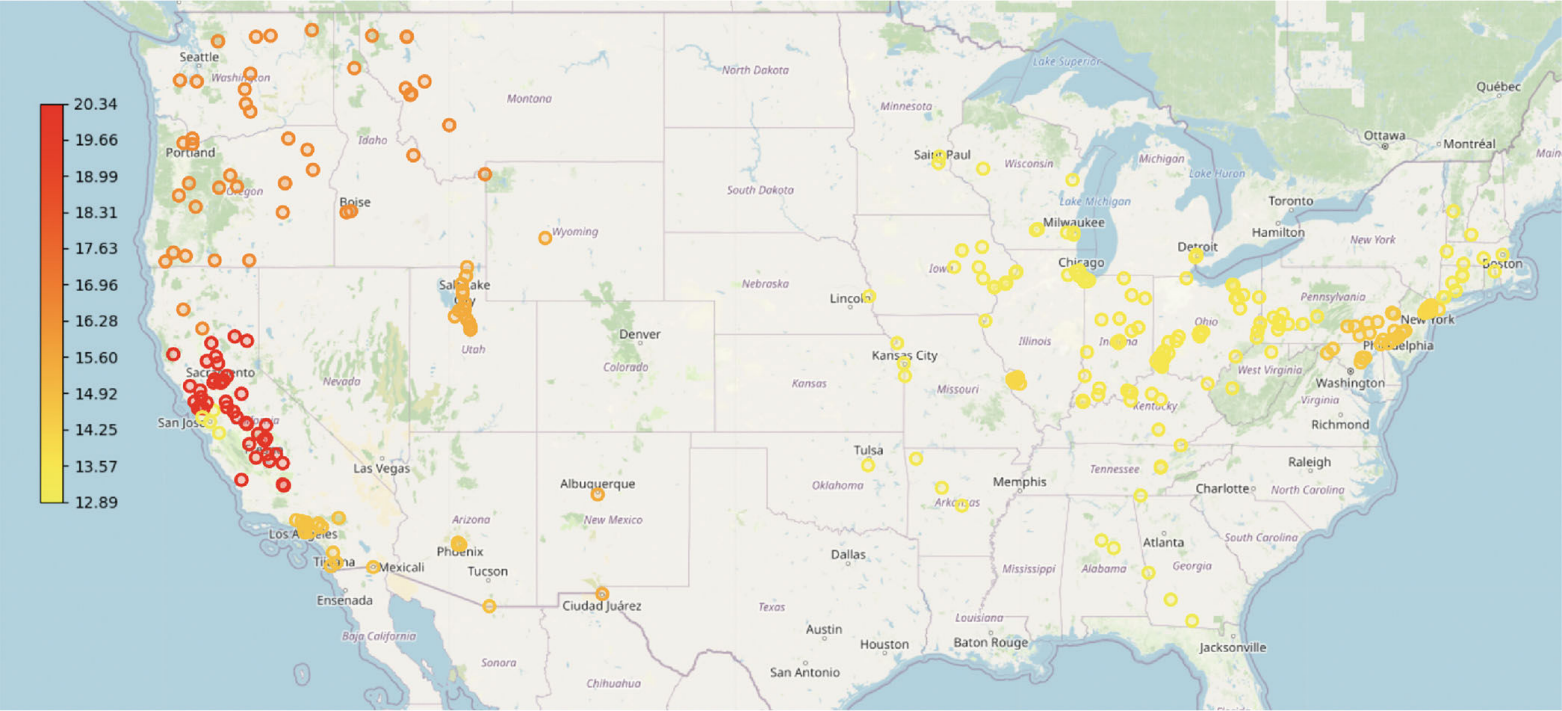
Average PM_2.5_ level for each clusters.

Furthermore, Figure 10 showcases the varying pollution levels across each cluster. Cities on the West Coast, especially in California, display higher pollution levels compared to those in the midland and on the East Coast. Among eastern cities, areas encompassing Philadelphia, New York, and St Louis show elevated PM_2.5_ values. This clustering result also aligns with the “Most Polluted City” rankings by the American Lung Association [19].

### 4.2 | U.S. Top Four Bank Clustering

In this study, we applied the DTC clustering algorithm to analyze branch-level bank data from the FDIC Annual Survey of Branch Office Deposits (Summary of Deposits), conducted annually on June 30. This survey is mandatory for all banks with branch offices and provides comprehensive data on the spatial distribution of banking networks. Our analysis focuses on the top four banks in the United States, which collectively account for approximately 50% of domestic deposits, making them a significant subject for study.

This analysis focuses on the state of Virginia from 2003 to 2023. Virginia’s diverse mix of metropolitan areas and rural counties provides an ideal testing ground to evaluate the algorithm’s ability to capture the geo-spatial distribution of banking networks across varying population densities and economic landscapes. While our primary analysis is limited to Virginia, this choice is not critical. Robustness checks expanding the analysis beyond Virginia’s borders show that the clustering results remain largely consistent.

The animation of the clustering results in each year in the Supporting Information highlights the stability of clusters in major metropolitan areas, including the Washington D.C. suburbs, Richmond, and Virginia Beach, over the two decades analyzed. These areas represent significant population centers where banks typically maintain large branch clusters, as expected. Conversely, rural areas in western Virginia reveal two notable changes in clustering patterns.

**FIGURE 11 |:**
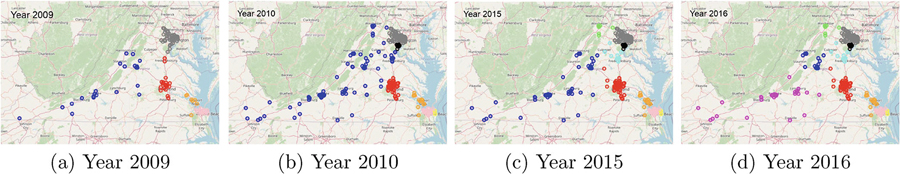
DTC clustering on top four banks in Virginia in the years 2009, 2010, 2015, and 2016.

First, as shown in Figure 11a,b, we observe a significant expansion of rural branch networks by the top four banks between 2009 and 2010. In the aftermath of the Great Recession (2008 – 2009), the U.S. government enacted stringent regulatory policies, such as the Dodd-Frank Wall Street Reform and Consumer Protection Act of 2010, which disproportionately burdened smaller banks. This hastened a trend of consolidation in the banking sector, leading to increased market concentration — a well-documented phenomenon in the economics literature. Our clustering analysis demonstrates that this consolidation involved the top banks acquiring local branches from smaller, financially stressed banks. Notably, this process appears to have occurred not gradually but through a concerted effort by the top four banks to acquire branches within a relatively short period.

Second, from Figure 11c,d, we observe a fragmentation of large rural branch clusters in western Virginia between 2015 and 2016. This timing coincides with the activities of the Virginia Growth and Opportunity (GO Virginia) Board, an economic development initiative that fosters collaboration among local governments, higher education institutions, private industry, and workforce development entities.^1^ In 2016, the board certified nine distinct GO Virginia regions, each characterized by shared economic development goals and interests. The clustering patterns post-2015 align closely with these regions. For example, Region Eight, including Harrisonburg and Staunton, corresponds to the Blue cluster in 2016 and beyond, and Region Two, including Roanoke and Lynchburg, corresponds to the Pink cluster in 2016 and beyond. These changes in clusters demonstrate how top banks may have realigned their branch networks based on these regional economic developments.

Upon closer examination, the fragmentation into smaller clusters appears to be driven by the closure of branches in small rural towns by top national banks. For example, Bank of America sold its Dillwyn branch to a smaller regional bank in 2015. Dillwyn, a small incorporated town with a population of fewer than 500 people, is located between Richmond and Lynchburg. This trend aligns with concerns raised by the Federal Reserve regarding the rise of banking deserts in low-income rural areas following the Great Recession.^2^ Banking deserts, as defined by the Federal Reserve, are areas where residents face limited access to financial services, often resulting in higher costs, difficulty obtaining loans, and increased reliance on predatory financial products.

Our clustering exercise yields two key insights:

- The emergence of banking deserts is potentially exacerbated by the retreat of top national banks from small towns. While the complete absence of banks in a banking desert is a significant concern, the withdrawal of major national banks can also severely limit access to financial services, creating additional challenges for affected communities. For instance, prior to 2015, deposits at the Dillwyn branch averaged $31.8 million, but following its sale by Bank of America, they dropped to $23.5 million — a notable reduction.
- The causes of banking deserts are multifaceted. Economic realignments within a state can drive endogenous adjustments to banking deposit networks, reshaping the distribution of financial services.

These findings are of considerable interest to economists and warrant further investigation to better understand the broader implications of banking consolidation and the geographic redistribution of branch networks.

## 5 | Conclusion and Future Work

This paper introduced the DTC algorithm, a novel framework designed to address the challenges of clustering data with irregular shapes, heterogeneous densities, and complex spatial domains. By integrating advanced density estimation, Delaunay triangulation, and a modified DBSCAN approach, DTC effectively overcomes the limitations of traditional clustering algorithms. Our numerical experiments and real-world applications demonstrate DTC’s versatility and robustness in identifying clusters across a wide range of scenarios, including datasets with adjacent, nested, and bridging clusters.

A key strength of the DTC algorithm lies in its triangulation-based density estimation method, which excels at capturing spatial relationships within complex domains. Furthermore, the DTC algorithm’s exceptional ability to address the “touching” problem, a common challenge in spatial clustering, positions it as a highly competitive alternative to other existing clustering methodologies. In the rapidly advancing fields of machine learning and pattern recognition, there is a growing demand for spatial clustering tools that can efficiently handle datasets with intricate and nonuniform structures. Responding to this demand, we have optimized the DTC algorithm by precomputing neighbors to eliminate redundant scanning, vectorizing operations for distances, areas, and edge lengths, and leveraging efficient data structures for faster access. These enhancements significantly reduce computational overhead, particularly when applied to large and high-dimensional datasets. Utilizing indexing libraries further enhances scalability by efficiently managing nearest-neighbor searches and spatial queries.

Moving forward, we aim to develop a triangulation-specialized indexing algorithm to further improve the speed and scalability of DTC. This advancement is expected to broaden the algorithm’s applicability across various domains within machine learning, enabling solutions for increasingly complex and diverse clustering challenges. The implementation of a faster and more scalable DTC algorithm has the potential to make a pivotal contribution to the field, addressing the rising complexity and size of datasets encountered in modern applications.

## Supplementary Material

Supp

Additional supporting information can be found online in the Supporting Information section.

## Figures and Tables

**TABLE 1:** Accuracy comparison table.

	Kernel K-mean (%)	HDBSCAN (%)	DBSCAN (%)	OPTICS (%)	DTC (%)
Aggregation dataset	86.29	82.74	86.42	84.77	99.62
A.K. Jain’s Toy Problem	78.55	80.70	65.15	98.93	100.00
Zahn’s Compound dataset	57.89	84.71	80.95	91.23	99.50
Noisy Circle	50.00	100.00	50.00	53.00	100.00
Aniso	66.67	92.00	100.00	83.33	100.00
Varied	67.77	87.00	81.00	94.00	94.00
Complex domain	66.66	94.00	88.00	63.00	99.00

## Data Availability

The data that support the findings of this study are available in multiple websites at https://github.com/MiichelleZhou/Density-and-Triangulation-based-Clustering. These data were derived from the following resources available in the public domain: Clustering basic benchmark, https://cs.joensuu.fi/sipu/datasets/; Scikit-learn Clustering data, https://scikit-learn.org/1.5/modules/clustering.html; Branch-level bank data, https://www.fdic.gov/analysis/bank-data-statistics; PM2.5 data, https://www.epa.gov/outdoor-air-quality-data/download-daily-data.

## Footnotes

1 https://govirginia.org/.

2 https://www.stlouisfed.org/publications/regional-economist/second-quarter-2017/banking-deserts-become-a-concern-as-branches-dry-up.
