# Supplementary material for: Triangulation-Based Spatial Clustering for Adjacent Data With Heterogeneous Density
Source: Stat Anal Data Min. Author manuscript; Available in PMC 2026 Apr 8. (PMC13055920; doi:10.1002/sam.70017)
Supplement: Supp [file NIHMS2156148-supplement-Supp.pdf]

Supplemental Materials for "Triangulation-based Spatial Clustering for Adjacent Data with Heterogeneous Density"

This supplementary document provides additional findings from the branch-level bank data analysis presented in Section 4.2 of the main article. Specifically, we illustrate the clustering results for each year from 2003 to 2023, as depicted in the animation shown in Figure S.1.

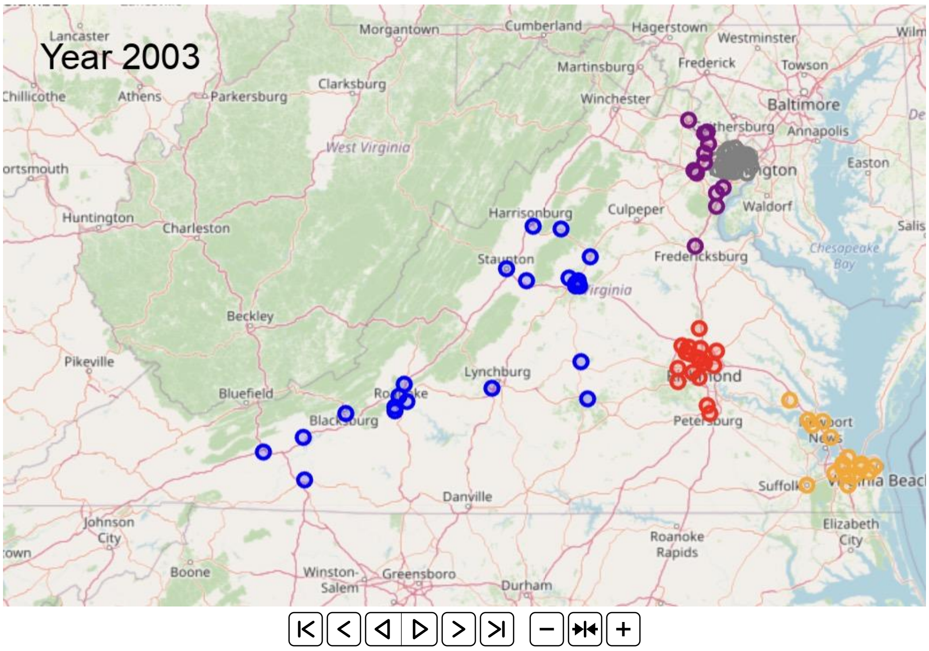

Figure S.1: Clustering of Top Four National Bank Branches in Virginia, 2003–2023
